# AAV9 Targets Cone Photoreceptors in the Nonhuman Primate Retina

**DOI:** 10.1371/journal.pone.0053463

**Published:** 2013-01-30

**Authors:** Luk H. Vandenberghe, Peter Bell, Albert M. Maguire, Ru Xiao, Tim B. Hopkins, Rebecca Grant, Jean Bennett, James M. Wilson

**Affiliations:** 1 Gene Therapy Program, Department of Pathology and Laboratory Medicine, University of Pennsylvania, Philadelphia, Pennsylvania, United States of America; 2 F. M. Kirby Center for Molecular Ophthalmology, Scheie Eye Institute, University of Pennsylvania, Philadelphia, Pennsylvania, United States of America; UNC Eshelman School of Pharmacy, United States of America; Xiao

## Abstract

Transduction of retinal pigment epithelial cells with an adeno-associated viral vector (AAV) based on serotype 2 has partially corrected retinal blindness in Leber congenital amaurosis type 2. However, many applications of gene therapy for retinal blindness rely on the efficient transduction of rod and cone photoreceptor which is difficult to achieve with first generation vector technology. To address this translational need, we evaluated rod and cone photoreceptor targeting of 4 novel AAV capsids (AAV7, AAV9, rh.64R1 and rh.8R) versus AAV2 and AAV8 in a foveated retina. Eyes of 20 nonhuman primates were injected subretinally in the proximity of the fovea. While numerous vectors efficiently transduced rods, only AAV9 targeted cones both centrally and peripherally efficiently at low doses, likely due to the abundance of galactosylated glycans, the primary receptor for AAV9, on cone photoreceptors. We conclude AAV9 is an ideal candidate for strategies that require restoration of cone photoreceptor function.

## Introduction

The normal human retina contains two main classes of light-sensing neurons: rod photoreceptors (PR), which are sensitive to dim light, and cone PR, which respond to bright light stimuli. Gene mutations hinder the function of either or both of these sets of cells, and lead to their degeneration and subsequent loss of vision. Over 200 different genes/loci are implicated in these types of blinding disorders (http://www.sph.uth.tmc.edu/retnet/disease.htm). Retinitis pigmentosa (RP) primarily affects rod PR but can result in secondary abnormalities of cones [1]. Cone and cone-rod dystrophies such as Stargardt's disease are characterized by a primary cone involvement, with possibly concomitant loss of rods [2]. Achromatopsia is associated with reduced or minimal cone function, and the complete form of this disorder is autosomal recessive in inheritance [3]. Age-related macular degeneration affects rods and cones centrally in the retina due to atrophy of the retinal pigment epithelium (RPE).

The normal arrangement and ratios of cone and rod photoreceptors across the retina are important variables affecting disease presentation. Only primates have a cone-rich macula and cone-only fovea; this region provides humans (and other primates) with fine visual resolution and color discrimination. Besides its involvement in retinal degenerative disease, the macula is vulnerable to damage from other genetic and environmental insults (e.g., age-related macular degeneration and diabetic retinopathy). While some non-primate retinas have regions of increased cone density (e.g., canine area centralis), none reflect the organization, set of color pigments, or high cone density as in primates. Cones are particularly sparse in rodent models of human retinal disease.

Multiple gene therapy strategies for inherited retinal degeneration are actively considered and have been tested in animal models, including: a) gene augmentation, in which a correct cDNA of the disease gene is introduced in the native cell type; b) ocular expression of a trophic factor geared to stall disease progression; c) gene knock-down of a toxic gene product in combination with gene augmentation; and d) re-sensitization of the remaining retinal cells to light [4]. In one promising method of re-sensitization of the retina, genetic reactivation of atrophic cones can be achieved by cone-targeted expression of halorhodopsin, a light-activated chloride pump isolated from *Archaea* [5].

Vectors based on AAV have shown distinct promise for *in vivo* applications of retinal gene therapy for PR degenerative disease. Vectors coated with different AAV capsid structures such as those derived from naturally occurring serotypes demonstrate dose-related tropism following subretinal injection [6]. All human applications of AAV gene transfer to the retina, and most other target organs, have utilized vectors based on serotype 2 (AAV2) [7], [8], [9]. Small and large animal studies demonstrate that AAV2 primarily targets the RPE following subretinal injection. AAV2-mediated transduction of RPE has achieved partial reconstitution of function in three different clinical trials for a severe, early onset form of RP termed Leber congenital amaurosis caused by a defect in RPE65. Whereas these trials rely on gene augmentation in the RPE, the majority of the other gene defects that can lead to blindness will require targeting of PR including rods and/or cones [10]. PR transduction is feasible with high-dose AAV2 vectors in canine, feline and primate animal models where it targets rods more efficiently than cones [6], [11], [12]. AAV5 targets PR more readily, but analogous to AAV2 also preferentially targets rods [13], though some level of cone and rod transduction was observed with the use of the human rhodopsin kinase promoter [14]. Indeed, studies using AAV5 with cone-specific promoters and at high dose did achieve functional rescue of achromatopsia (rod monochromacy) in a dog [15] and dichromatism (red-green color blindness) in an NHP model [16]. AAV7 and AAV8 are more effective than AAV5 in PR targeting in mouse [17], [18]. In dogs, subretinally injected AAV8 demonstrated significant transduction of the neuroretina including PRs [19]. AAV8 pig studies reflect similar findings with some but limited cone transduction [20]. Data from our previous NHP study demonstrated that AAV8 was markedly more efficient at targeting rod PR than AAV2 at all doses studied. Partial cone transduction was achieved but only at elevated doses of 10^11^ GC [6].

What determines the tropism and pharmacology of AAV serotypes in the retina remains largely unknown, although studies in other therapeutic target organs noted that serotypes interact differentially with entry and post-entry cellular determinants of transduction. AAV9 was recently found to use terminal galactose on cell-surface bound glycans as its receptor *in vitro* and *in vivo* [21]. AAV2 is known to utilize heparin sulfate proteoglycans as its primary receptor for cellular recognition [22]. Viral entry of AAV1, 4, 5, and 6 is initiated by sialylated glycoproteins [23], [24].

The distinct properties of AAV serotypes in terms of tropism and dosage thresholds in the retina and other organs motivated us to explore other natural AAV variants derived from novel viral clades identified in a biomining effort in our laboratory from human and NHP tissues [25], [26]. In this study, six promising capsids representing different clades were selected for evaluation in NHP including AAV2, AAV7, AAV8, AAV9, rh.8R and rh.64R1 in order to quantitatively assess RPE, rod and cone transduction.

## Results

Cynomolgus macaques, 2–3 years of age, were injected subretinally with 10^9^ or 10^10^ GC of AAV.CMV.eGFP packaged with the respective capsids. A total of 40 eyes from 20 animals were injected with vector and subjected to experimental analysis. Informative doses to evaluate vector tropism were established in previous AAV dose-ranging studies with AAV2 and AAV8 [6]. Injections were generally superior-temporal the details of which are summarized in **Table 1**. In some eyes, the subretinal exposure area extended over the fovea. Most injections were uneventful, however in eight eyes, surgical complications were noted (**Table 1**). The most significant complications occurred with injections in the vicinity of the fovea. In two eyes, a fistula developed through the fovea and vector leaked through the macular hole. In addition to the retinal complications, hyphema (anterior chamber blood) developed prior to injection at the time of paracentesis in another two eyes, although these did not obscure the retina during the injection procedure. The other complications involved unintentional deposition of vector in areas outside the subretinal space. Of note, the surgical procedure in the animal studies described here is not the same used in humans, where standard 3 port pars plana vitrectomy (without paracentesis) is performed. The NHP injection procedure is modified to take into account the unique surgical anatomy of these smaller animals and the desire to reduce anesthesia time.

**Table 1:** Design, surgical and ophthalmoscopic observations.

					RIGHT								LEFT							
	animal	weight		duration	dose	SR	IV	notes	GFP Score			OD	dose	SR	IV	notes	GFP Score			OD
	ID	(kg)		(days)	10^x^	(%)	(%)		1w	1mo	3mo		10^x^	(%)	(%)		1w	1mo	4mo	
**AAV7**	C21332	2.30	♂	127.00	10	100	0		0	3	4	+	9	100	0	2x	0	2−	2−	−
	C21410	2.60	♂	127.00	10	100	0	A,F	0	4−	4+F	+	9	90	10		0	1	2+	−
	C21418	2.35	♂	128.00	9	100	0	JF	0	2−	2−	−	10	100	0	JF	0	3+	4	+
	C21427	2.45	♂	135.00	10	50	0	F	0	4	4	−	9	100	0	F	0	0	3−	−
	C21429	2.25	♂	135.00	9	*0*	*100*	*IV*	*0*	*0*	*1*	−	10	100	0	F	0	2+	2F	−
**AAV9**	C21361	2.30	♂	146.00	10	100	0	F	0	4	4F	+	9	100	0	F	0	1	2−	−
	C21382	2.35	♂	146.00	10	100	0	B, F	0	4−	4	+	9	*15*	*0*	*SRPE,M*	*0*	*0*	*2+*	−
	C21387	2.50	♂	147.00	10	*30*	*70*	*IV*	*0*	*4*	*4*	+	9	100	0		0	2+	2−	−
	C21388	2.75	♀	147.00	10	100	0		0	3	4	−	9	100	0		0	2	3	−
	C21390	2.35	♀	149.00	10	100	0	A,F	0	4	4	+	9	*10*	*60*	*SRPE*	*0*	*0*	*0*	−
**rh64R1**	C21366	2.35	♀	153.00	10	100	0	F	0	4	4F	NA	9	100	0	JF	0	2	2	−
	C21369	2.30	♀	153.00	10	*50*	*50*	*F*	*0*	*1*−	*3+*	+	9	*60*	*0*	*SRPE,F*	*0*	*2*	*0*	−
	C21376	2.10	♀	154.00	10	100	0	F	0	4	4	+	9	100	0	F	0	2+	2	−
	C21379	2.30	♀	154.00	10	*100*	*0*	*B,F*	*4*	*3*	*4*	+	9	50	50		0	0	3	−
	C21380	2.50	♀	156.00	10	*100*	*0*	*2x,A,M,F*	*0*	*3*−	*4*	−	10	100	0	F	0	4	4	+
**rh8R**	C21336	2.30	♀	118.00	10	100	0	2x	0	4	4+F	−	9	100	0	2x	0	2−	3−	−
	C21339	2.35	♀	118.00	10	100	0		2	4−	3+F	+	9	100	0		0	3	2−	−
	C21342	2.30	♀	119.00	10	100	0		2	4	4+F	+	9	100	0		0	2	2+	−
	C21355	2.25	♀	119.00	10	100	0		0	4	4+F	+	9	100	0		0	2	3+	+
	C21360	2.40	♀	121.00	10	100	0		1	4	4+	+	9	100	0		1	2+	3+	+

**Figure 1:**
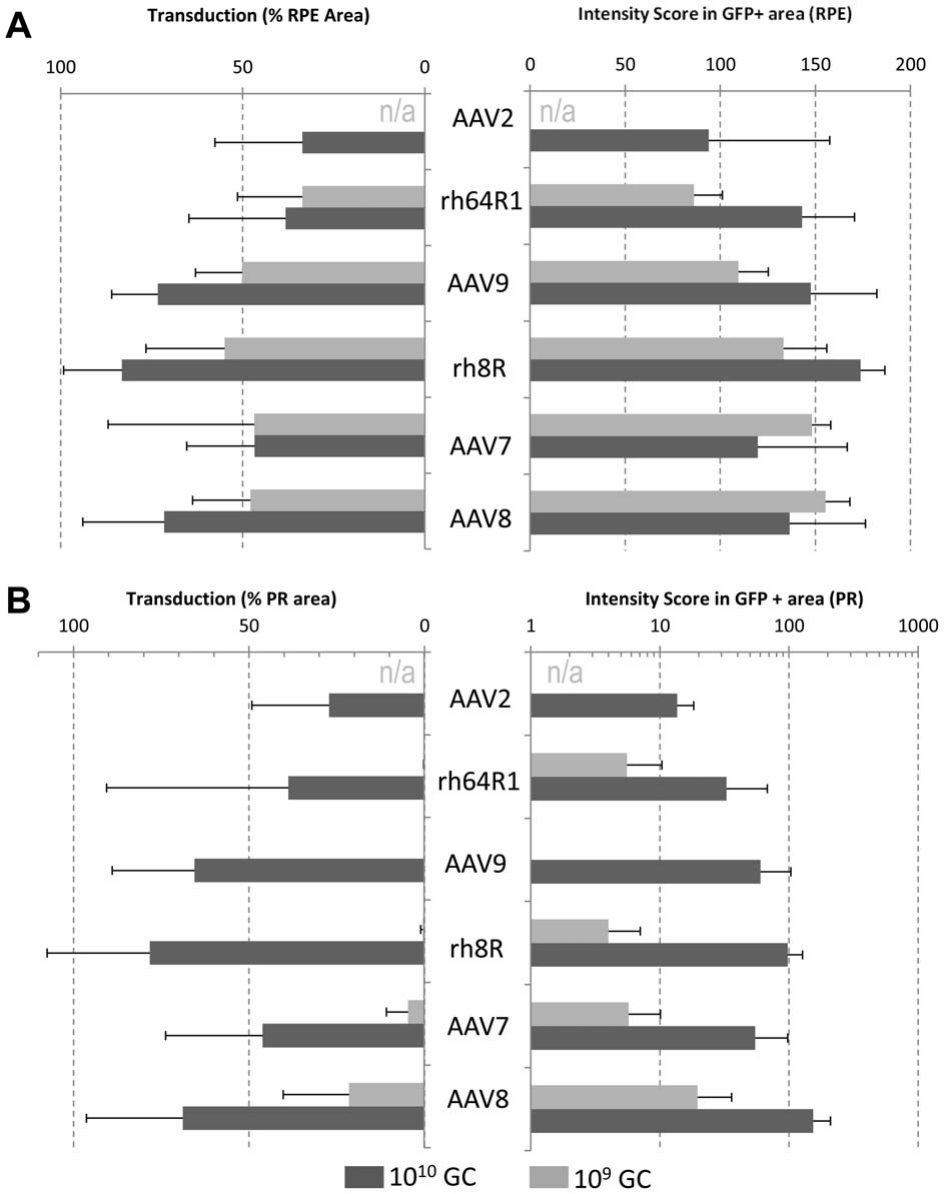
Quantitative analysis of tropism and transgene expression levels in the NHP eye. Cynomolgus macaques were injected with AAVs expressing 10^9^ or 10^10^ GC per eye. Following necropsy at 4–5 month post injection, retinas were sectioned and analyzed for direct fluorescence. Data from a morphometric analysis in the RPE (A) and PR (B) layers is presented with the relative area of transduction on the left and an intensity scoring on the right. AAV2 and AAV8 data are historical data from an analogous, previously reported study [6] [a 10^9^ GC injection was not performed for AAV2 (n/a)]. Eyes for which the injection failed as noted in Table 1 were excluded from this analysis. Data is presented as average and standard deviation.

Cells with italicized font are excluded from the quantitative post mortem analysis shown in Fig. 1 due to injection issues as highlighted in the notes column; % SR and IV reflect estimates of vector deposit in subretinal and vitreal spaces respectively; GFP Score ranges from 0 (no visible expression) to 4 (broad and intense GFP) and reflects a subjective composite of both intensity and area of transduction as observed by indirect ophthalmoscopy; F: foveal; B: blood; A: visible air bubble; 2x: injection procedure was interrupted following sclerotomy and restarted making use of the initial sclerotomy a second time due to technical concerns; SRPE: subRPE or choroidal injection; JFL: juxtafoveal; M: macular hole; SR: subretinal; IV: intravitreal; ID: identification number; GFP: green fluorescent protein; OD: optic disc.

Animals were followed for general well-being and retinal health throughout the study. The breadth, intensity and onset of the retinal GFP expression were monitored by indirect ophthalmoscopy (**Table 1**). Clinicopathologic correlates were evaluated for 4–5 months following injection after which animals were euthanized and tissue was harvested for extensive and detailed histological analysis.

Indirect ophthalmoscopy assessed intensity and distribution of retinal GFP expression during the in-life phase of the study. The composite score, which incorporated intensity and area of transduction, ranged from a low of 0 to a high of 4. Expression peaked at 1 month and was stable for the duration of the study. More than 50% of eyes had detectable GFP in the optic disc at the high dose. Remarkably, only two eyes in the low dose presented GFP in the optic disc and both were rh.8R-transduced (**Table 1**). Histological sections from several relevant retinal regions of each eye were analyzed including the injection area, the fovea and optic disc. The retina is organized into regions circumferential to the foveola/fovea including the parafovea, perifovea, and the periphery (**Figure S1**). To quantitatively assess vector targeting, morphometric analysis for GFP in PR (without rod/cone differentiation) and RPE was performed in the most distal regions from the fovea including the perifovea and periphery. **Figure 1** summarizes these data for the 4 candidate capsids and includes those from similarly designed studies with AAV2 and AAV8 [6] in terms of the relative area of transduction and GFP intensity in transduced areas. RPE transduction was found to be variable yet efficient with all experimental serotypes at either dose suggesting that transduction of RPE by AAV at these doses is not determined by dose or capsid (**Figure 1A**). At the high dose, PRs were broadly and intensely transduced with most AAV types, though some serotype-dependency was noted. Due to the abundance of rods in the regions evaluated here, data in **Figure 1B** largely captures rod transduction with cones contributing only marginally. Substantially lower transduction was observed at the 10^9^ GC dose, with a clear serotype-dependent permissivity of PRs. Whereas AAV9, rh.64R1 and rh.8R achieve very limited rod PR transduction at 10^9^ GC, AAV7 or AAV8 result in GFP expression in 5% and 22% of rod PR within the region exposed to vector, respectively, at levels of expression similar to AAV2 at a 10-fold higher dose (**Figure 1B**).

**Figure 2:**
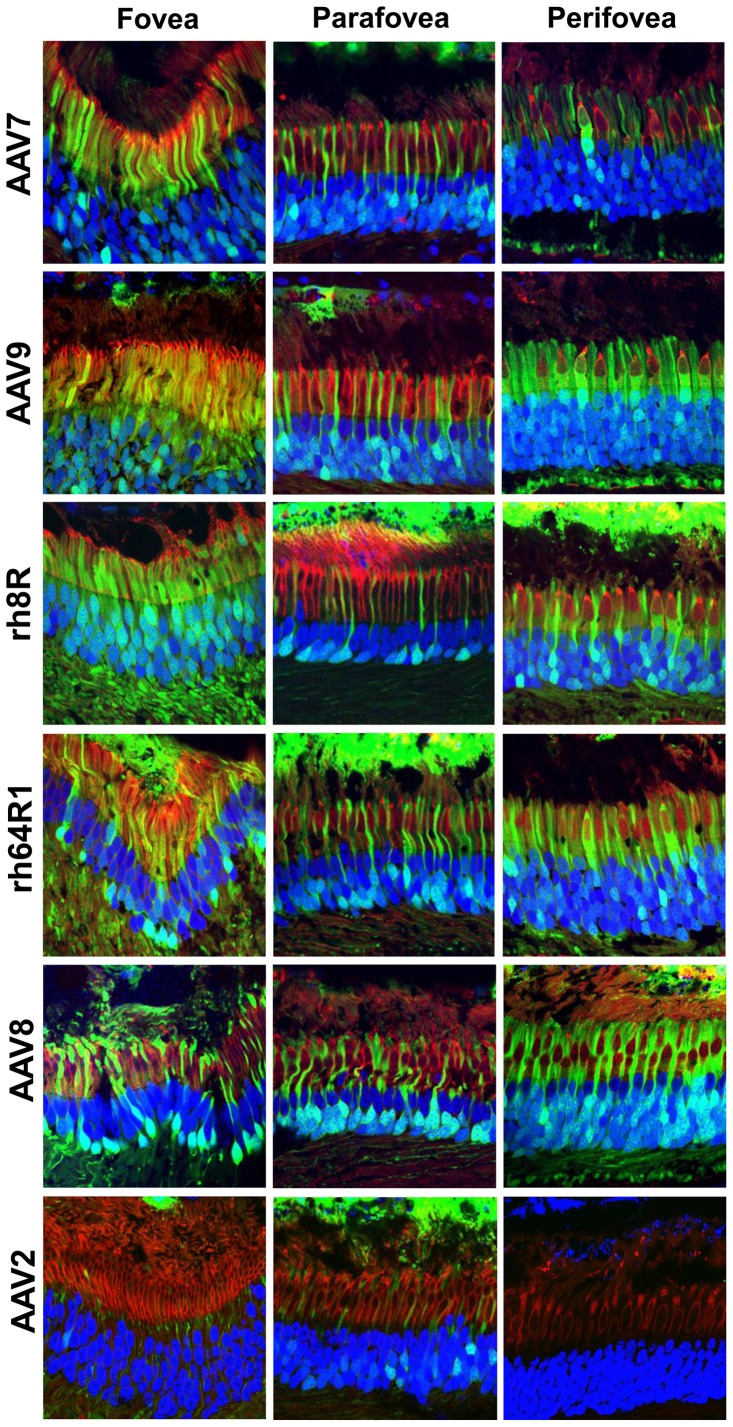
AAV cell targeting in the fovea, parafovea and perifovea. Histological sections were stained with DAPI (blue) and peanut agglutinin (red), a lectin specific for terminal galactose residues prevalent on cone PR, and finally visualized for GFP (green) by direct fluorescence. The foveal, parafoveal and perifoveal regions were identified based on topology and cone density. Perifoveal areas were chosen in a region between 1.3 and 1.9 mm from the fovea. Within the subretinal injection area, cone transduction in the peripheral retina was similar in efficiency to that in the perifovea.

To study in greater detail the relative targeting of rods and cones, we expanded the analyses to include the cone-only foveola, and the concentric ring around the foveola named the parafovea, as well as the surrounding cone-enriched perifovea, and the retinal periphery (**Figure S1**). Histological analysis provided in Figure 2, illustrates that across serotypes, foveal cones are more readily transduced as compared to extrafoveal cones. High dose of AAV9 (10^10^ GC) however appeared to achieve higher levels of cone transduction in the fovea and the perifovea (**Figure 2** **)** than other vectors, including AAV2 and 8 at the even higher dose of 10^11^ GC [6]. A surprising observation with all AAVs tested, including AAV9, was that only limited cone transduction was observed in the parafoveal region, even in the presence of RPE and rod PR transduction (**Figure 2**).

**Figure 3:**
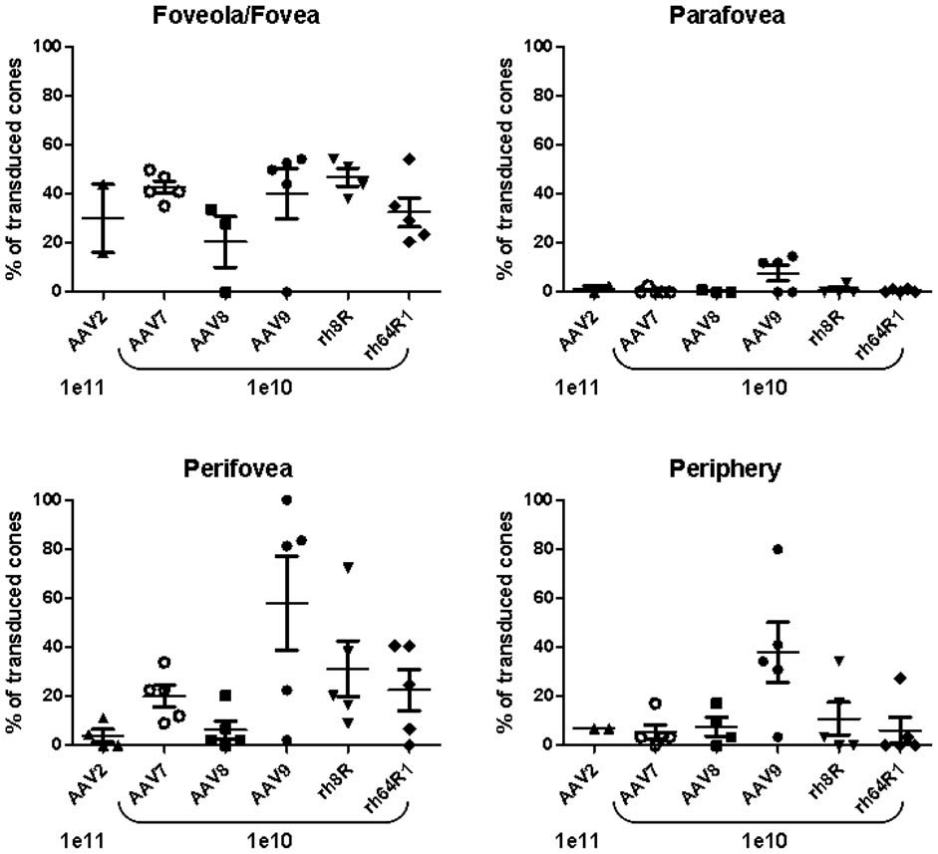
Quantitative assessment of cone photoreceptor transduction across the nonhuman primate retina. For each eye and for each of the foveal, parafoveal, perifoveal and peripheral retina regions, GFP positive cones were counted on histological sections. Shown is the percentage of the average number of GFP-positive cones relative to the average total cone number for each eye and region. Each eye for which viable sections were available was included in this analysis, including those for which the injection was suboptimal or problematic and the bleb may not have extended across the fovea/parafovea.

A quantitative assessment of cone transduction of all vectors at a moderate dose of 10^10^ GC compared to AAV2 at a 10-fold higher dose illustrated similar levels of transduction in the fovea for all serotypes ranging from 20% for AAV8 and approximately 40% for AAV9 and rh.8R (**Figure 3**). In some eyes, no foveal cone transduction was found, indicating that vector was likely not able to reach this retinal region following injection. Conversely, some injections were not noted to include the fovea however transduction in this area was noted, due to either diffusion beyond the bleb or, more likely, expansion of the bleb following surgery and monitoring after the animal became mobile after anesthesia (**Table 1**). As evidenced from the histological data in **Figure 2**, parafoveal transduction is minimal with AAV9 however still superior to all other AAVs tested. In the perifoveal macular region and beyond in the peripheral retina, cone transduction was achieved robustly by AAV9, and to a lesser extent rh.8R and rh.64R1. AAV7 is fairly weak in its ability to target cone PR but still outperforms AAV8 and AAV2 in this respect. In ideal conditions of vector delivery to the subretinal space, AAV9 was able to target over 80% of cones in the perifoveal macula (**Figure 3**).

## Discussion

In summary, we show that AAV9 uniquely targets cone PRs at high efficiency. This property may be due to the abundance of terminal galactose, the cognate receptor for AAV9 [21], [27], on cone PR in vertebrates including humans [28], as we confirmed by the specific and high level of the lectin peanut agglutinin (PNA) [28] staining of cone PR in our studies (**Figure 2**). The findings here in which a dose-related, quantitative assessment of vector targeting is made for all cells lining the subretinal space provide a first step toward understanding the pharmacodynamics of AAV in this setting. It is apparent from our data and others that most AAVs efficiently transduce the RPE at low to moderate doses. However, where rod PR transduction is highly efficient with AAV7 and particularly AAV8 at lower doses, AAV9, rh.8R and rh.64R1 do not perform quite as well (**Figure 1B**). Conversely, cone targeting with AAV9, rh.8R and rh.64R1 is superior to that of AAV7 and particularly AAV8 (**Figure 2** **and** **3**). We speculate that these findings are due to an intricate combination of differential receptor usage, saturation of vector binding sites on the surface of the cell type of interest and particle trapping in the glycan matrix within the subretinal space. Our observation that transducing parafoveal cones is challenging, even with a highly efficient cone targeting vector such as AAV9, may be a function of some of these factors. Future studies will have to be designed to determine whether injection procedure and/or dose may be able to overcome this hurdle. Ultimately these data will contribute to a deeper pharmacological understanding of the use of AAV in the emerging clinical field of gene therapy treatments for inherited and acquired forms of blindness.

Our comprehensive analysis of a number of AAV vectors based on different capsid structures in NHP retinas provides directly useful information for treating a large spectrum of inherited retinopathies following subretinal injection. Virtually any AAV capsid including that from serotype 2 efficiently targets RPE which would be sufficient in a limited number of diseases, the most celebrated being LCA due to RPE65. A majority of the remaining disorders require high level transduction of rod PR such as X-linked RP due to RPGR mutations; RP due to PDE6B mutations or rhodopsin mutations; and LCA due to lebercilin mutations. Our studies suggest that AAV8 is best suited for these diseases based on efficiency of rod transduction in NHP retina. However, AAV9 may be best suited for strategies targeting cones with endocrine survival factors, functional rescue of central vision using optogenetic restoration of vision in cones, or for gene augmentation for inherited retinopathies which require transduction of cones, such as achromatopsia and Stargardt disease.

## Experimental Procedures

### Animals, Injection and Follow-up

Cynomolgus macaques were treated and cared for at the Nonhuman Primate Research Program facility of the Gene Therapy Program of the University of Pennsylvania (Philadelphia, PA) during the study. Anterior chamber fluid was tapped prior to injection to relieve intraocular pressure. The studies were performed in accordance with study protocols approved by the Environmental Health and Radiation Safety Office, the Institutional Biosafety Committee, and the Institutional Animal Care and Use Committee of the University of Pennsylvania. At the time of enrollment, all animals in the study had serum neutralizing antibody titer to AAV of less than 1/20. Injections were complicated by hyphemas caused by the anterior segment tap in a few eyes (**Table 1**). The study length was between 119 and 156 days at which time the eyes were collected and fixed for histology (**Table 1**). Injection procedure, clinical and ophthalmoscopic follow-up are as previously described [6]. Fundus photos were taken with a hand-held Kowa fundus camera.

### Vectors

AAV vectors were manufactured and purified from cell lysates by PennVector (http://www.med.upenn.edu/gtp/vectorcore/) by triple transfection in HEK293 cells as previously described [29]. The transgene plasmid encoded an early cytomegalovirus promoter (CMV), the enhanced green fluorescent protein and a woodchuck hepatitis virus post-transcriptional regulatory element, and the bovine growth hormone poly-adenylation (bGH) signal. Vector preparations were assayed for quality by multiple assays including TaqMan quantitative PCR with primers and probes directed towards bGH for genome (GC) titration (which is repeated independently 3 times for NHP studies), whole protein analysis by SDS-PAGE for purity, and endotoxin determination with <20 EU/ml as a lot release criterion.

### Histology and Morphometry

Histological sectioning was performed analogously as described for the previously published AAV2 and AAV8 study [6]. GFP morphometry in RPE and ONL was performed with ImageJ software (Rasband 1997–2006; National Institutes of Health, Bethesda, MD, http://rsb.info.nih.gov/ij/) on only those NHP eyes injected with the entire dose and without concerns related to the injection (**Table 1**). For 5 representative sections of each injected eye, two separate measuring lines were drawn through the RPE and the outer nuclear layer. Images and the brightness in the green channel were quantified per pixel as a value between 0 and 255. Background levels were established per pixel from a section from an uninjected retina. Percent of transduced area was determined as an average per eye and per group by determining the relative number of pixels above background as compared to the total number of pixels within the injected area. Intensity was established by averaging per eye and per group the intensity score of the RPE and ONL per section. Intensity scoring per positive area was performed in the NHP to represent level of relative expression of positively transduced areas and calculated per eye by averaging intensity values only when GFP signal exceeded background level.

Determination of percentage of GFP-positive cones was performed by GFP-positive cone counts per retina region (foveola/fovea, para-, perifovea, periphery). Images were taken from sections corresponding to the plane shown in Figure S1 at identical exposure time but variable gain setting to visualize both strong and weak GFP expression. Typically two and sometimes three images were recorded per region, for the foveola only one picture could be taken due to the small size of this structure. The images were recorded so that the retina was in a horizontal position within the image and its length equivalent to 235 μm. Cones were considered positive for GFP expression if they were visually clearly recognizable as both GFP-positive and as cone PR and if they had a minimum intensity value at least 3-fold over the background as measured in an untransduced area (usually within the choroidea) within the same image. The intensities were determined with ImageJ software. For every serotype and region the average number of GFP-positive cones per 235 μm section was calculated. The eyes evaluated were injected at a dose of 10^10^ vector genome copies except for AAV2 with 10^11^ genome copies (10^10^ gave too low expression levels to be comparable with other serotypes). One eye (C21366 OD) was excluded due to lack of expression, likely due to the fact that the injection area was not included in the injection. In some eyes (18173 ODR, 18204 ODR, 18144 ODR, 18168 ODR, C21360 ODR) the section containing the fovea could not be exactly determined, in this case cone transduction in the fovea and parafovea was not evaluated but a section parallel to the fovea section was used to count transduced cones in the perifovea and periphery. Total cone numbers per section for each region were determined by counts from sections stained with rhodamin-labeled peanut agglutinin (PNA), a cone-specific stain. To this end images were taken for every region from PNA-stained sections obtained from five to eleven eyes and the number of all cones was counted per 235 μm section and averaged per region. The percentage of GFP-positive cones was then determined by calculating the ratio of GFP+ cones to total cone counts per section for each region.

## Supporting Information

Figure S1 **Topology sampling for cone tropism study.** Mapping of the foveal, parafoveal and perifoveal regions was done based on measured distance from the fovea and morphological hallmarks. Specifically, the fovea and foveola are located along the slopes or in the foveal pit which has the highest cone density. The parafovea, at a distance of 350–500 μm from the foveal center, is located on the foveal rim, i.e., the circular rim surrounding the fovea where the retina is thickest, largely due to a thicker retinal ganglion cell layer. The perifovea was located 1.3–1.9 mm from the fovea, and finally the peripheral retina, at 3.3–4.3 mm, which is part of the extrafoveal macula. D, optic disc; F, foveola/fovea; I, injection site. Images show cones in red (PNA stain) and nuclei in blue (DAPI).
(PDF)

## References

[1] Milam AH, Li ZY, Fariss RN (1998) Histopathology of the human retina in retinitis pigmentosa. Prog Retin Eye Res 17: 175–205. 9695792 10.1016/s1350-9462(97)00012-8

[2] Hamel CP (2007) Cone rod dystrophies. Orphanet J Rare Dis 2: 7. 17270046 10.1186/1750-1172-2-7 PMC1808442

[3] Pang JJ, Alexander J, Lei B, Deng W, Zhang K, et al (2010) Achromatopsia as a potential candidate for gene therapy. Adv Exp Med Biol. 664: 639–646. 10.1007/978-1-4419-1399-9_73 PMC3608407 20238068

[4] Jacobson SG, Cideciyan AV (2010) Treatment possibilities for retinitis pigmentosa. N Engl J Med 363: 1669–1671. 20961252 10.1056/NEJMcibr1007685

[5] Busskamp V, Duebel J, Balya D, Fradot M, Viney TJ, et al (2010) Genetic reactivation of cone photoreceptors restores visual responses in retinitis pigmentosa. Science 329: 413–417. 20576849 10.1126/science.1190897

[6] Vandenberghe LH, Bell P, Maguire AM, Cearley CN, Xiao R, et al (2011) Dosage thresholds for AAV2 and AAV8 photoreceptor gene therapy in monkey. Sci Transl Med 3(88): 88ra54. 10.1126/scitranslmed.3002103 PMC5027886 21697530

[7] Bainbridge JW, Smith AJ, Barker SS, Robbie S, Henderson R, et al (2008) Effect of gene therapy on visual function in Leber's congenital amaurosis. N Engl J Med 358: 2231–2239. 18441371 10.1056/NEJMoa0802268

[8] Hauswirth WW, Aleman TS, Kaushal S, Cideciyan AV, Schwartz SB, et al (2008) Treatment of Leber congenital amaurosis due to RPE65 mutations by ocular subretinal injection of adeno-associated virus gene vector: short-term results of a phase I trial. Hum Gene Ther 19: 979–990. 18774912 10.1089/hum.2008.107 PMC2940541

[9] Maguire AM, Simonelli F, Pierce EA, Pugh EN Jr, Mingozzi F, et al (2008) Safety and efficacy of gene transfer for Leber's congenital amaurosis. N Engl J Med 358: 2240–2248. 18441370 10.1056/NEJMoa0802315 PMC2829748

[10] den Hollander AI, Black A, Bennett J, Cremers FP (2010) Lighting a candle in the dark: advances in genetics and gene therapy of recessive retinal dystrophies. J Clin Invest 120: 3042–3053. 20811160 10.1172/JCI42258 PMC2929718

[11] Bennett J, Maguire AM, Cideciyan AV, Schnell M, Glover E, et al (1999) Stable transgene expression in rod photoreceptors after recombinant adeno-associated virus-mediated gene transfer to monkey retina. Proc Natl Acad Sci U S A 96: 9920–9925. 10449795 10.1073/pnas.96.17.9920 PMC22311

[12] Bainbridge JW, Mistry A, Schlichtenbrede FC, Smith A, Broderick C, et al (2003) Stable rAAV-mediated transduction of rod and cone photoreceptors in the canine retina. Gene Ther 10: 1336–1344. 12883530 10.1038/sj.gt.3301990

[13] Beltran WA, Boye SL, Boye SE, Chiodo VA, Lewin AS, et al (2010) rAAV2/5 gene-targeting to rods: dose-dependent efficiency and complications associated with different promoters. Gene Ther 17: 1162–1174. 20428215 10.1038/gt.2010.56 PMC2914811

[14] Boye SE, Alexander JJ, Boye SL, Witherspoon CD, Sandefer K, et al (2012) The human rhodopsin kinase promoter in an AAV5 vector confers rod and cone specific expression in the primate retina. Hum Gene Ther 23: 1101–1115. 22845794 10.1089/hum.2012.125 PMC3472519

[15] Komaromy AM, Alexander JJ, Rowlan JS, Garcia MM, Chiodo VA, et al (2010) Gene therapy rescues cone function in congenital achromatopsia. Hum Mol Genet 19: 2581–2593. 20378608 10.1093/hmg/ddq136 PMC2883338

[16] Mancuso K, Hauswirth WW, Li Q, Connor TB, Kuchenbecker JA, et al (2009) Gene therapy for red-green colour blindness in adult primates. Nature 461: 784–787. 19759534 10.1038/nature08401 PMC2782927

[17] Allocca M, Mussolino C, Garcia-Hoyos M, Sanges D, Iodice C, et al (2007) Novel adeno-associated virus serotypes efficiently transduce murine photoreceptors. J Virol 81: 11372–11380. 17699581 10.1128/JVI.01327-07 PMC2045569

[18] Lebherz C, Maguire A, Tang W, Bennett J, Wilson JM (2008) Novel AAV serotypes for improved ocular gene transfer. J Gene Med 10: 375–382. 18278824 10.1002/jgm.1126 PMC2842078

[19] Stieger K, Colle MA, Dubreil L, Mendes-Madeira A, Weber M, et al (2008) Subretinal delivery of recombinant AAV serotype 8 vector in dogs results in gene transfer to neurons in the brain. Mol Ther 16: 916–923. 18388922 10.1038/mt.2008.41

[20] Mussolino C, della Corte M, Rossi S, Viola F, Di Vicino U, et al (2011) AAV-mediated photoreceptor transduction of the pig cone-enriched retina. Gene Ther 18: 637–645. 21412286 10.1038/gt.2011.3 PMC3131697

[21] Bell CL, Vandenberghe LH, Bell P, Limberis MP, Gao GP, et al (2011) The AAV9 receptor and its modification to improve in vivo lung gene transfer in mice. J Clin Invest 121: 2427–2435. 21576824 10.1172/JCI57367 PMC3104778

[22] Summerford C, Samulski RJ (1998) Membrane-associated heparan sulfate proteoglycan is a receptor for adeno-associated virus type 2 virions. J Virol 72: 1438–1445. 9445046 10.1128/jvi.72.2.1438-1445.1998 PMC124624

[23] Kaludov N, Brown KE, Walters RW, Zabner J, Chiorini JA (2001) Adeno-associated virus serotype 4 (AAV4) and AAV5 both require sialic acid binding for hemagglutination and efficient transduction but differ in sialic acid linkage specificity. J Virol 75: 6884–6893. 11435568 10.1128/JVI.75.15.6884-6893.2001 PMC114416

[24] Wu Z, Miller E, Agbandje-McKenna M, Samulski RJ (2006) Alpha2,3 and alpha2,6 N-linked sialic acids facilitate efficient binding and transduction by adeno-associated virus types 1 and 6. J Virol 80: 9093–9103. 16940521 10.1128/JVI.00895-06 PMC1563919

[25] Vandenberghe LH, Breous E, Nam HJ, Gao G, Xiao R, et al (2009) Naturally occurring singleton residues in AAV capsid impact vector performance and illustrate structural constraints. Gene Ther 16: 1416–1428. 19727141 10.1038/gt.2009.101 PMC2795093

[26] Gao G, Vandenberghe LH, Alvira MR, Lu Y, Calcedo R, et al (2004) Clades of Adeno-associated viruses are widely disseminated in human tissues. J Virol 78: 6381–6388. 15163731 10.1128/JVI.78.12.6381-6388.2004 PMC416542

[27] Shen S, Bryant KD, Brown SM, Randell SH, Asokan A (2011) Terminal N-linked galactose is the primary receptor for adeno-associated virus 9. J Biol Chem 286: 13532–13540. 21330365 10.1074/jbc.M110.210922 PMC3075699

[28] Blanks JC, Johnson LV (1984) Specific binding of peanut lectin to a class of retinal photoreceptor cells. A species comparison. Invest Ophthalmol Vis Sci 25: 546–557. 6715128

[29] Vandenberghe LH, Xiao R, Lock M, Lin J, Korn M, et al (2010) Efficient serotype-dependent release of functional vector into the culture medium during adeno-associated virus manufacturing. Hum Gene Ther 21: 1251–1257. 20649475 10.1089/hum.2010.107 PMC2957237

