# Correction: AAV9 Targets Cone Photoreceptors in the Nonhuman Primate Retina

**DOI:** 10.1371/annotation/64b90996-4634-4c63-b737-634397b0b445

**Published:** 2013-08-08

**Authors:** Luk H. Vandenberghe, Peter Bell, Albert M. Maguire, Ru Xiao, Tim B. Hopkins, Rebecca Grant, Jean Bennett, James M. Wilson

A project funding the seventh author was incorrectly omitted from the Funding Statement. The second sentence of the Funding Statement should read: "This work was also supported by the Foundation Fighting Blindness (LHV, JB), its CHOP-PENN Pediatric Center for Retinal Degenerations (JB), Transdisciplinary Award Program in Translational Medicine and Therapeutics (TAPITMAT) from the University of Pennsylvania (LHV, JB), Research to Prevent Blindness, the Paul and Evanina Mackall Foundation Trust at Scheie Eye Institute, the F. M. Kirby Foundation (JB) and Treatrush, European Commission (JB)." 

