# Supplementary figures and images for: AAV9 Targets Cone Photoreceptors in the Nonhuman Primate Retina
Source: PLoS One. 2013 Jan 30;8(1):e53463. doi: 10.1371/journal.pone.0053463 (PMC3559681; doi:10.1371/journal.pone.0053463)

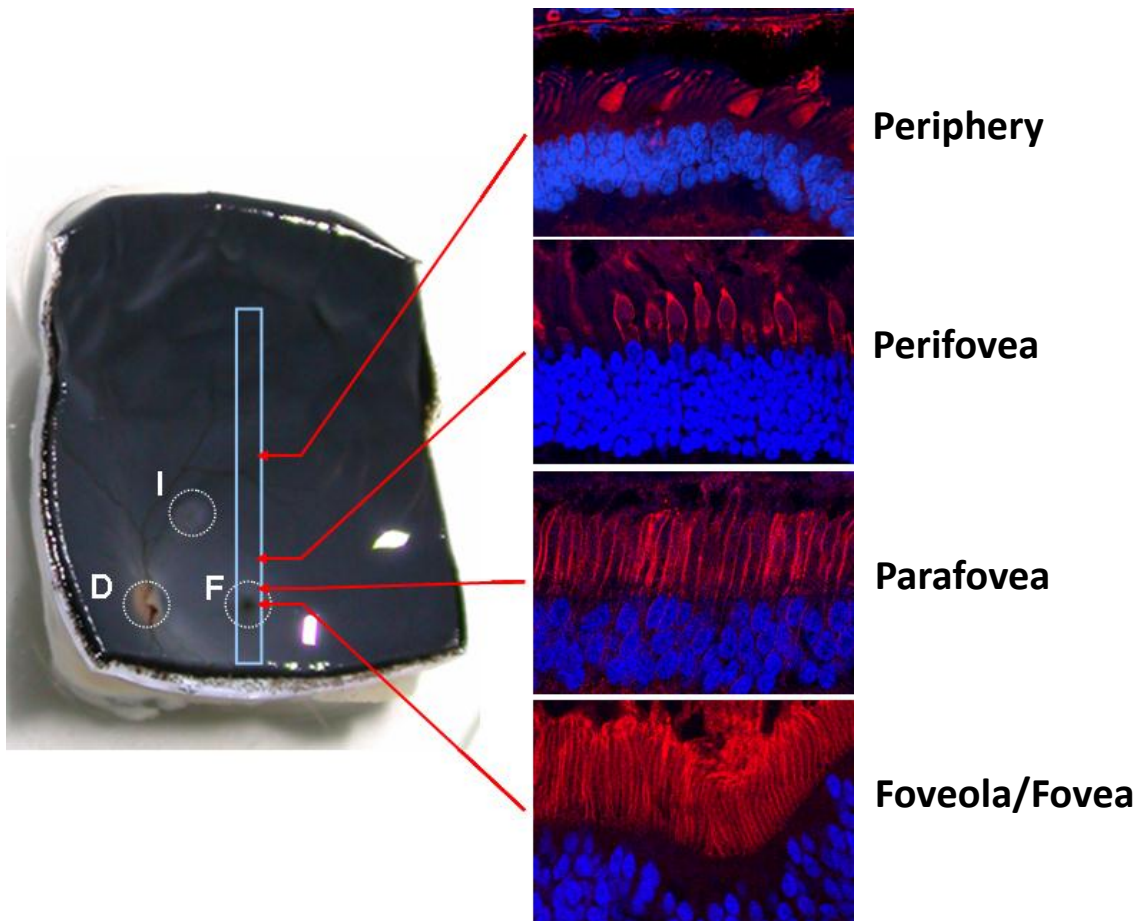

Supplement: Figure S1 — Topology sampling for cone tropism study. Mapping of the foveal, parafoveal and perifoveal regions was done based on measured distance from the fovea and morphological hallmarks. Specifically, the fovea and foveola are located along the slopes or in the foveal pit which has the highest cone density. The parafovea, at a distance of 350–500 μm from the foveal center, is located on the foveal rim, i.e., the circular rim surrounding the fovea where the retina is thickest, largely due to a thicker retinal ganglion cell layer. The perifovea was located 1.3–1.9 mm from the fovea, and finally the peripheral retina, at 3.3–4.3 mm, which is part of the extrafoveal macula. D, optic disc; F, foveola/fovea; I, injection site. Images show cones in red (PNA stain) and nuclei in blue (DAPI). (PDF) [file pone.0053463.s001.pdf]
